# Factors associated with health-related quality of life 1 year after COVID-19: a cross-sectional study in Sweden

**DOI:** 10.48101/ujms.v130.13260

**Published:** 2025-12-19

**Authors:** Marta A. Kisiel, Helena Janols, Adriana-Maria Hiller, Christoffer Forsell, Claes Kock, Carl-Johan Neiderud, Gabriel Westman, James Janani, Magnus Ekström, Andrei Malinovschi, Christer Janson

**Affiliations:** a Department of Medical Sciences, Environmental and Occupational Medicine, Uppsala University, Uppsala, Sweden; b Department of Medical Sciences, Section of Infectious Diseases, Uppsala University, Uppsala, Sweden; c Department of Clinical Sciences in Malmö, Lund University, Occupational and Environmental Medicine, Lund, Sweden; d Department of Respiratory Medicine, Allergology and Palliative Medicine, Institution for Clinical Sciences in Lund, Lund University, Lund, Sweden; e Department of Medical Sciences, Clinical Physiology, Uppsala University, Uppsala, Sweden; f Department of Medical Sciences, Respiratory, Allergy and Sleep Research, Uppsala University, Uppsala, Sweden

**Keywords:** Health-related quality of life, COVID-19, patient-reported outcome measures, EQ-5D-5L, persistent symptoms, work ability index, six-minute walking test, dyspnoea-12 (D-12) questionnaire

## Abstract

**Aims:**

The main aim of the present study was to identify factors influencing health-related quality of life (HRQoL), as measured by EQ-5D-5L, in individuals 1 year after the acute phase of COVID-19.

**Methods:**

This cross-sectional study included 75 participants (51% females), 1 year after confirmed SARS-CoV-2 infection (76% hospitalised). Associations between HRQoL, measured by patient-reported outcome measures (PROMs): EQ-5D-5L, and factors of interest, including persistent symptoms, comorbidities, work ability index (WAI), lung function, six-minute walking test (6MWT), and dyspnoea-12 (D-12) questionnaire, were assessed by an analysis of variance (ANCOVA) model (adjusted by various factors of interest) with post hoc pairwise comparison.

**Results:**

In the ANCOVA, lower HRQoL was significantly associated with not having a university education (mean difference [MD] with 95% confidence interval [CI]: 0.115 [0.013–0.217]), a higher number of persistent symptoms (for 10 vs. 1–3 symptoms: −0.153 [−0.291, −0.015]), lower work ability (for poor vs. excellent: −0.283 [−0.445, −0.120]), any comorbidity (0.077 [0.015–0.138]), and affective distress in the D-12 (0.257 [0.096–0.417]). No significant sex differences in HRQoL and the level of care at the acute infection were shown. In descriptive analysis, lower HRQoL was significantly associated with age under 55, sick leave, more dyspnoea in D-12, and poorer work ability.

**Conclusions:**

Work ability, comorbidities, persistent symptoms, and affective distress were associated with lower HRQoL at 1-year follow-up after COVID-19. No significant differences in HRQoL were observed between sexes. Our study highlights the need to address HRQoL in post-COVID-19 rehabilitation and broader public health planning.

## Introduction

COVID-19, caused by severe acute respiratory syndrome coronavirus-2 (SARS-CoV-2), is a zoonotic infection declared a pandemic between 11 March 2020 and 5 May 2023. According to the World Health Organization (WHO), as of 27 April 2025, there have been 777,745,434 confirmed cases and 7,095,349 deaths globally attributed to COVID-19, whereas 1,458 only during the last month (1). Although most infected individuals recover, a significant portion experiences persistent symptoms beyond 12 weeks following an acute infection, a disorder known as post-COVID-19 condition or long COVID-19 (2). A wide range of symptoms characterises this condition, where the most commonly reported symptoms include fatigue, dyspnoea, cognitive impairment, insomnia, myalgia, and cardiovascular issues (3). An association between these symptoms and reduced health-related quality of life (HRQoL) and working ability has been demonstrated (4). However, factors modifying the long-term HRQoL remain less understood, highlighting the need for further investigation to better address its determinants and impact.

HRQoL is a multidimensional concept encompassing an individual’s physical, mental, social, and daily functioning regarding health conditions (5). It plays a crucial role in supporting individuals and is essential for overall recovery, community well-being, and collective prevention. While numerous studies have examined the HRQoL in individuals with post-COVID-19 condition, few have incorporated patient-reported outcome measures (PROMs) and clinical objective measures over a long follow-up period. This gap highlights the need for comprehensive studies integrating multiple perspectives to capture the full impact of post-COVID-19 symptoms on the HRQoL.

The aetiology of decreased HRQoL in individuals following COVID-19 infection is poorly understood. According to empirical evidence, there is a bidirectional link between mental and physical health (6). Behavioural studies from the USA have shown that experiencing COVID-19 increases psychological distress and anxiety (7). Additionally, international experts have suggested that the pandemic contributed to various stressors, including fear resulting from social isolation, limited access to healthcare, and uncertainties related to employment (8). These factors may vary depending on national response strategies. For example, Sweden did not enforce a mandatory lockdown, unlike many countries. Instead, it recommended voluntary social distancing and travel limitations, which may have influenced the psychological and social burden of the pandemic differently.

HRQoL, including in post-COVID-19 populations, is frequently assessed using the PROM known as the EuroQol 5-Dimension 5-Level (EQ-5D-5L). The strength of EQ-5D-5L lies in its availability of reference value sets, including those specific to the general population of Sweden (9). This instrument assesses five dimensions of health: mobility, self-care, usual activities, pain/discomfort, and anxiety/depression, each with five levels of severity. It also provides a global health assessment through the EQ visual analogue scale (EQ VAS) and a utility score reflecting the health state from the general population’s perspective (10). In our previous research, we demonstrated the capability of EQ-5D-5L to capture key persistent symptoms following COVID-19, such as fatigue and memory/concentration issues (11). Despite its utility, further research is needed to investigate the broader factors that influence HRQoL in this patient population.

The main aim of the present study was to identify factors influencing HRQoL, as measured by EQ-5D-5L, in individuals 1 year after the acute phase of COVID-19.

## Material and methods

### Study design

This study was a cross-sectional analysis conducted within the COMBAT post-COVID study at Uppsala University, aimed at evaluating the long-term health consequences following COVID-19 (4, 12, 13). The current analysis included individuals with a positive Polymerase chain reaction (PCR) for SARS-CoV-2 at COVID-19 onset, who were followed up 1 year after the infection. At follow-up, participants underwent lung function testing and weight and height measurement. They completed questionnaires including sociodemographic characteristics, persistent symptoms, HRQoL with EQ-5D-5L, work ability with work ability index (WAI), and dyspnoea-12 (D-12) at the Department of Respiratory, Allergy and Sleep Research.

This study followed the Helsinki Declaration and was approved by the Swedish Ethical Review Authority (Dnr 2021-01891 and Dnr 2022-01261-01). All participants gave written informed consent to participate.

### Study population

A total of 566 non-hospitalised participants diagnosed with COVID-19 between March and December 2020 at the Department of Infectious Diseases, Uppsala University Hospital, were invited to participate in the study. Invitations were distributed via REDCap (a web-based application for surveys and databases) or postal mail (12). These participants were asked to complete a questionnaire collecting sociodemographic data and self-reported information on persistent symptoms 12 months after infection.

In addition, 180 individuals hospitalised for COVID-19 at the same department were invited to a structured follow-up between December 2020 and May 2021. Of these, 42 were excluded due to being over the age of 67 (*n* = 40) or due to death before follow-up (*n* = 2).

The recruitment process was based on all available eligible patients at the Department of Infectious Diseases during the defined period and level of care, rather than random sampling. Participation was voluntary among those invited.

**Figure 1:**
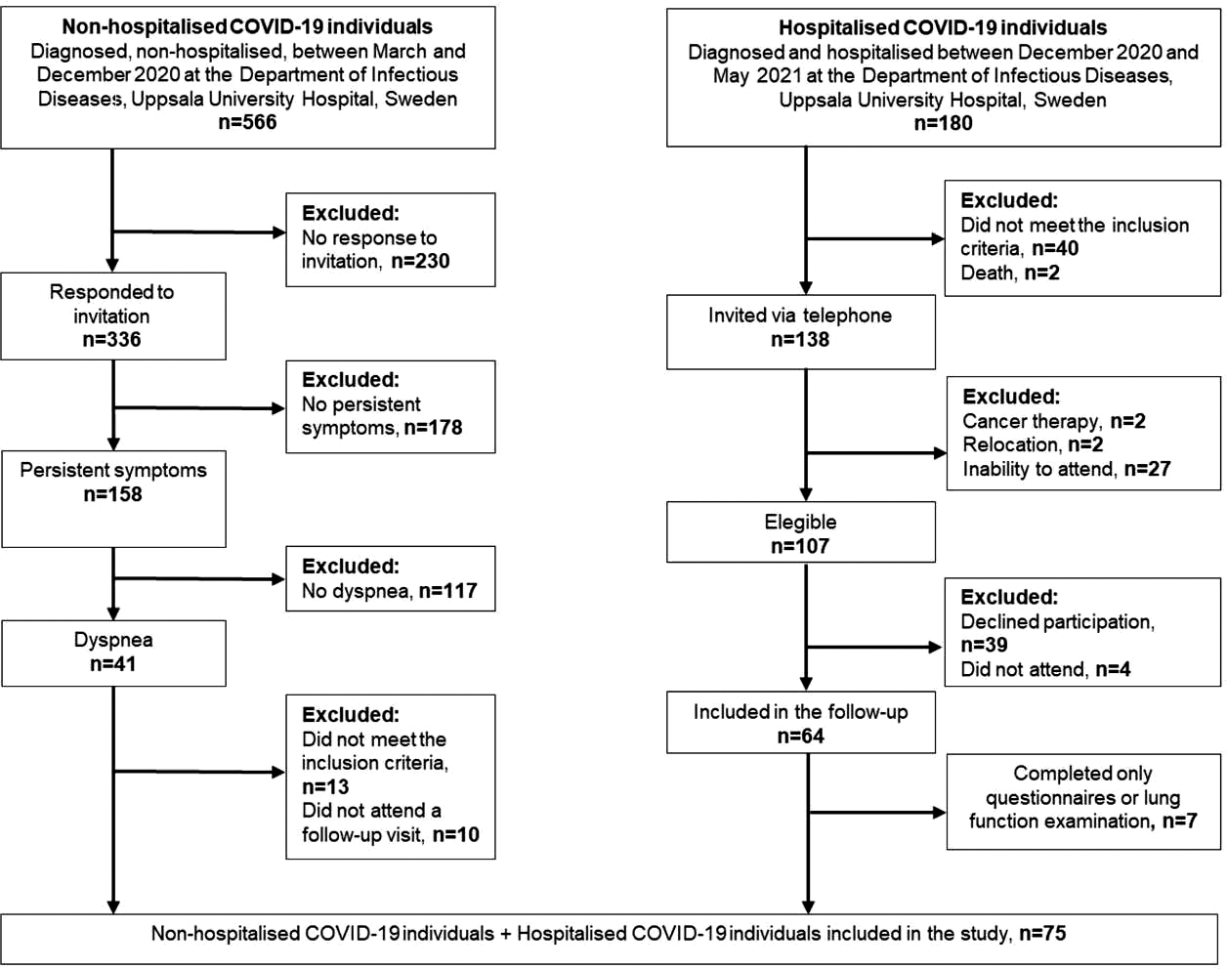
Flowchart of the study population.

The flowchart (Figure 1) illustrates the final study sample, stratified by level of care during the acute phase (non-hospitalised vs. hospitalised).

### Primary outcome: HRQoL

HRQoL was assessed using the EQ-5D-5L, which consists of five dimensions: mobility, self-care (ADL, activity of daily living), usual activities, pain/discomfort, and anxiety/depression. Each dimension is evaluated for severity using a five-point scale: 1 = no problems, 2 = slight problems, 3 = moderate problems, 4 = severe problems, and 5 = extreme problems (10). Additionally, the questionnaire included a visual analogue scale (EQ-VAS), where responders rated their health on a vertical axis ranging from zero, ‘the worst imaginable condition’, to 100, indicating ‘the best imaginable condition’. The EQ-5D-5L was presented as a health profile by combining the responses into a five-number profile, with higher values reflecting worse HRQoL (10). Using a scoring algorithm, the EQ-5D-5L health states were converted into a single utility score algorithm, the EQ-5D-5L health states were converted into a single utility score. This study used the Swedish value set and scoring algorithm to calculate utility scores (9).

### Factors of interest

The factors of interest for the present study were persistent symptoms after COVID-19, work ability, dyspnoea, physical performance, and pulmonary function.

### Persistent symptoms

The participants were asked in the questionnaire to indicate whether they still experienced any of the listed persistent symptoms that appeared after their COVID-19 contraction a year before: dyspnoea, muscle/joint pain, headache, smell/taste impairment, cough, sore throat, runny nose, chest pain, heart palpitations, memory/concentration problems, sleep problems, fatigue, vertigo, depression/anxiety, skin problems, gastrointestinal (GI) problems (including stomach pain and diarrhoea) 1 year after the infection onset.

### Work ability

The WAI questionnaire includes a list of diseases and seven occupational health characteristics. WAI covers seven items: item 1, current work ability compared with lifetime best, 0–10 points; item 2, workability about the demands of the job, 2–10 points; item 3, number of current diseases diagnosed by a physician, 1–7 points; item 4, estimated work impairment due to diseases, 1–6 points; item 5, sick leave during the past year, 1–5 points; item 6, own prognosis of work ability two years from now, 1, 4, or 7 points; and item 7, mental resources, 1–4 points. Items 2, 3, and 7 comprise 2, 14, and 3 sub-items.

The final WAI score was calculated as the total unweighted score covering the WAI’s seven dimensions or indicators. The combination of dimension values results in a total WAI score that can range from 7 (unable to work) to 49 (full workability). Based on this score, an individual was classified into standard workability categories of excellent (WAI 44–49), good (WAI 37–43), moderate (WAI 28–36), and poor (WAI 27 or less) (14). The WAI was not calculated in the retired participants.

The first dimension of the WAI, referred to as the work ability score (WAS) index, measures current working capacity compared with lifetime best. To interpret WAS results, the inventors of this method suggested using the same type of categorisation as for the WAI, that is, poor (0–5 points), moderate (6–7), good (8–9), and excellent (10).

### Dyspnoea-12

Dyspnoea was assessed using the dyspnoea-12 (D-12) questionnaire. The D-12 instrument includes 12 descriptors evaluated on a 4-point scale, such as none (score 0), mild (score 1), moderate (score 2), or severe (score 3), resulting in a total score from 0 to 36, where a higher score corresponds to more severe breathlessness. The first seven items constitute a physical domain assessing whether the breath does not go in all the way, the patient cannot get enough air, feels short of breath, or has difficulty catching breath, and the breathing requires more work, is uncomfortable, or exhausting. The remaining five items constitute an emotional domain describing whether the breathing is distressing, irritating, or makes the patient feel depressed, miserable, or agitated. The range for the D-12 total score is 0–36: 0–21 for the physical score and 0–15 for the affective score. Higher D-12 scores indicate worse breathlessness (15). In the Swedish validation study of D-12, the minimal clinical importance difference (MCID) for patients with chronic obstructive lung disease (COPD) was reported to be 2.7 units for the total score and 1.4 and 1.2 units, respectively, for the D-12 physical and affective domains (16).

### Physical performance

The six-minute walking test (6MWT) is widely used in clinical care to evaluate an individual’s physical performance (assessed as the walk distance during 6 min) and prognosis. The 6MWT was conducted according to the ATS standards (17). The participants self-reported their intensity of breathlessness and leg discomfort at the end of each 6MWT using a modified Borg 0–10 category-ratio (CR10) scale. Predicted per cent for 6MWT (m) was assessed based on the algorithm validated in a previous study (18). A walk distance < 80% of the predicted value was considered abnormally low.

### Pulmonary function

Each participant underwent spirometry and lung diffusion capacity for carbon monoxide (DL_CO_) measurements using a Jaeger Master Screen PFT (Vyaire, Mettawa, IL, US). In dynamic spirometry, the following parameters were assessed: forced expiratory volume in one second (FEV_1_), forced vital capacity (FVC), and the ratio of FEV_1_ to FVC. At least three acceptable and reproducible manoeuvres were performed. Additionally, D_LCO_ was assessed. Pulmonary function tests adhered to the American Thoracic Society/European Respiratory Society guidelines (19). Determinations of FEV_1_, FVC, FEV_1_/FVC, and D_LCO_ below the lower limit of normal (LLN) corresponded to a z-score < −1.645, using the reference values of the Global Lung Function Initiative. Abnormal D_LCO_ was defined asDL_CO_ < LLN and assessed by the ATS/ERS guidelines.

### Other covariates

This study included data on educational level (university or not university), working status (currently working, on sick leave, retired and other), smoking status (current, ex-, never), daily using of Swedish snuff (being a moist, smokeless tobacco product that is placed under the upper lip), pre-existing comorbidities diagnosed/confirmed by a doctor before COVID-19 (hypertension, heart disease, lung disease [asthma or COPD], diabetes mellitus, depression/anxiety, chronic pain, ongoing immunological treatment, previous cancer treatment, hypo/hyperthyroidism). Body mass index (BMI) in kg/m^²^ was divided into four groups according to the WHO: underweight (<18.5 kg/m²), normal weight (18.5–24.9 kg/m²), overweight (25.0–29.9 kg/m²), and obesity (≥30.0 kg/m²). Additionally, the infection severity at onset was added in accordance to WHO Clinical Progression Scale between 0 and 10, whereas 0 is uninfected, 1 asymptomatic, 2 symptomatic, independent, 3 symptomatic, assistance needed, 4 hospitalised, no oxygen therapy, 5 hospitalised, oxygen by mask or nasal prongs, 6 hospitalised, oxygen by NIV or high flow, 7–9 intubation and mechanical ventilation, and 10 is dead. Patients discharged directly from the emergency room were deemed symptomatic and independent, and were assigned a score of 2.

### Statistical analyses

The statistical analyses were conducted using the R program version 4.2.3, with a statistical significance defined as a two-tailed *P* < 0.05. Basic characteristics of the participants were presented as mean with standard deviation (SD) or median with interquartile range (IQR) for continuous variables, and as absolute values (*n*) and percentages (%) for categorical variables. The difference between the two study groups was assessed using Fisher’s exact tests (for categorical variables) and *t*-tests (for continuous variables). The missing data were <5% per variable, as reported in Supplementary Table 1. The *eq5d* R package calculated the utility score of EQ-5D-5L. The differences across the EQ-5D-5L domains and between sexes for non-hospitalised and hospitalised participants were presented.

We performed an ANCOVA with a fixed factor, age to determine which factor of interest was associated with the EQ-5D-5L utility score (HRQoL). The main model included all relevant factors of interest: sex, smoking, BMI, comorbidity, number of persistent symptoms, fatigue, and WAI. D-11 and 6MWT were conducted only in the hospitalised group, with all included variables available (participants with missing variables were not included in the model), as non-hospitalised individuals lack D-12 and 6MWT. This model included *n* = 38. To capture association in all study participants, we performed a secondary ANCOVA model not including D-12 and 6MWT, and therefore included *n* = 59. In both models for each factor, we first report the ANCOVA *P*-value; when significant, we performed Tukey post hoc pairwise comparisons to quantify direction and magnitude (mean differences [MDs] and 95% confidence intervals [CIs]). The model framework was adapted from a previously studied COVID-19 population (20). The post hoc pairwise comparison was performed using Tukey’s HSD test and the glht function from the R package multcomp.

We prespecified the model factors and avoided stepwise variable selection to prevent model-selection bias and to retain prespecified confounders. The smaller sample in Model 2 reflects the study design: it is limited to hospitalised patients for whom 6MWT and D-12 scores were available; these measures were not collected in non-hospitalised participants. Consequently, Model 2 includes fewer observations.

## Results

### Characteristics of the study participants

This study included 75 participants, 51% female (the second column in Table 1). In all, 84% of participants reported persistent symptoms 1 year after COVID-19, whereas the most commonly reported symptoms were fatigue, dyspnoea, and memory/concentration problems (Supplementary Table 2). The most common comorbidities were hypertension and depression/anxiety. Most participants had normal lung function, but a minority had normal BMI. HRQoL was not significantly different between hospitalised (*n* = 57, 40% female) and non-hospitalised (*n* = 18, 83% female) participants. There was no significant difference between females and males in the EQ-5D-5L, EQ-VAS, or WAI (Supplementary Table 3).

**Table 1:** The distribution of mean ± SD of EQ-5D-5L utility score and mean ± SD of EQ-VAS across the subcategories of all factors of interest in the 75 study participants.

Factors of interest	*n* (%)	EQ-5D-5L utility score Mean ± SD	*P*	EQ VAS Mean ± SD	*P*
**Age** ≤ 55 years old > 55 years old	36 (48) 39 (52)	0.894 ± 0.173 0.913 ± 0.116	0.577	66.306 ± 17.887 76.154 ± 15.108	**0.012**
**Sex** Female Male	38 (51) 37 (49)	0.903 ± 0.156 0.904 ± 0.137	0.969	71.632 ± 17.408 71.216 ± 17.054	0.917
**Education level** No university University	25 (33) 50 (67)	0.880 ± 0.208 0.915 ± 0.107	0.448	72.20 ± 19.044 71.04 ± 16.263	0.796
**Smoking status** Current and ex- Never	26 (35) 49 (65)	0.922 ± 0.072 0.894 ± 0.174	0.337	70.654 ± 15.399 71.837 ± 18.105	0.767
**Any comorbidity** Yes No	53 (71) 22 (29)	0.893 ± 0.158 0.928 ± 0.114	0.296	71.925 ± 17.051 70.227 ± 17.626	0.704
**Hospitalisation** Yes No	57 (76) 18 (24)	0.893 ± 0.166 0.935 ± 0.044	0.100	71.228 ± 18.571 72.056 ± 11.790	0.825
**Number of symptoms** 0 1–3 4–6 7–9 10	12 (16) 27 (36) 14 (19) 9 (12) 13 (17)	0.973 ± 0.333 0.929 ± 0.088 0.905 ± 0.131 0.821 ± 0.175 0.843 ± 0.244	0.086	88.333 ± 8.876 75.185 ± 15.031 70.143 ± 12.751 55.000 ± 16.202 60.769 ± 15.791	**<0.001**
**BMI in kg/m**^2^ Normal 18.5–24.9 Overweight 25–29.9 Obesity ≥ 30	10 (14) 27 (38) 34 (48)	0.923 ± 0.061 0.950 ± 0.056 0.869 ± 0.194	0.094	74.500 ± 14.804 76.667 ± 15.993 67.118 ± 18.117	0.085
**D_LCO_** Normal < LLN	59 (86) 10 (16)	0.905 ± 0.113 0.963 ± 0.045	**0.008**	70.881 ± 16.011 76.500 ± 17.803	0.368
**WAI total score** Poor Moderate Good Excellent	7 (10) 16 (24) 28 (41) 17 (25)	0.636 ± 0.319 0.870 ± 0.115 0.942 ± 0.046 0.972 ± 0.035	**<0.001**	46.429 ± 15.999 66.562 ± 10.119 73.821 ± 15.847 82.353 ± 15.219	**<0.001**
**Index WAS** Poor Moderate Good Excellent	9 (13) 16 (23) 36 (51) 10 (14)	0.679 ± 0.287 0.898 ± 0.056 0.941 ± 0.086 0.969 ± 0.026	**<0.001**	55.556 ± 21.858 63.438 ± 13.505 75.611 ± 14.472 84.000 ± 13.081	**<0.001**
*6MWT predicted ≥ 80% < 80%	47 (92) 4 (8)	0.920 ± 0.093 0.614 ± 0.458	0.274	73.404 ± 16.521 48.750 ± 33.260	0.235
*D-12 total < MCID ≥ MCID	41 (79) 11 (21)	0.933 ± 0.096 0.815 ± 0.156	**0.034**	74.878 ± 16.900 60.455 ± 16.801	**0.023**
*D-12 affective < MCID ≥ MCID	41 (85) 7 (15)	0.911 ± 0.127 0.758 ± 0.317	0.250	72.609 ± 17.911 59.286 ± 22.991	0.185
*D-12 physical < MCID ≥ MCID	40 (77) 12 (23)	0.928 ± 0.098 0.840 ± 0.161	0.096	74.750 ± 16.639 62.083 ± 18.520	**0.049**

SD: standard deviation; BMI: body mass index; D-12: dyspnoea-12 questionnaire; DL_CO_: diffusing capacity of the lung for carbon monoxide; FEV₁: forced expiratory volume in 1 second; FVC: forced vital capacity; GI: gastrointestinal; LLN: lower limit of normal; WAI: work ability index; WAS: work ability score; WHO-scale: World Health Organization Progression Severity Score scale; 6MWT: six-minute walking test; MCID: minimal clinical important difference.

* Not performed in non-hospitalised participants.

Bold values indicate statistically significant results (*p* < 0.05).

### Health-related quality of life

In the descriptive analysis, the participants under age 55 years, on sick leave, with various persistent symptoms, particularly fatigue, depression/anxiety, and those experiencing more dyspnoea, measured by D-12 total score, reported significantly poorer HRQoL (lower EQ-5d-5L utility score and/or EQ-5D). HRQoL was also considerably lower in those reporting poorer work ability, measured by WAI (Table 1 and Supplemental Table 2).

**Figure 2:**
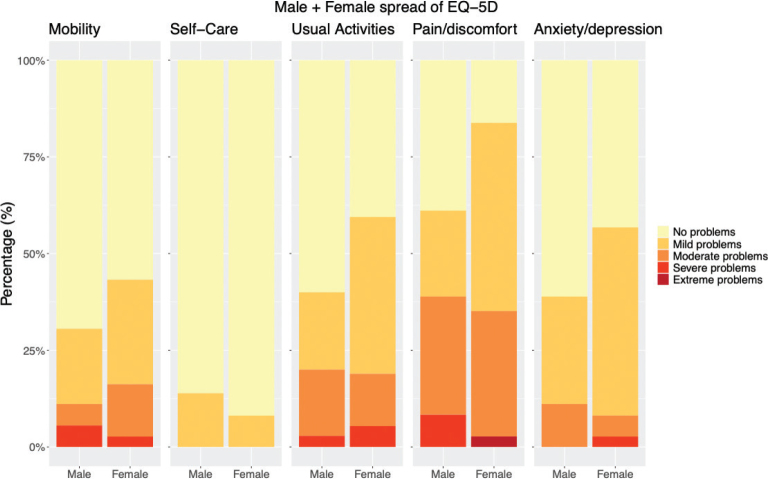
Visual distribution of male and female participants across the EQ-5D-5L dimensions.

More male participants reported no problems across all dimensions except for self-care. Pain and discomfort were the domains most frequently reported as causing severe issues in males and extreme difficulties in females (Figure 2).

### Association between the HRQoL and the factors of interest

The ANCOVA main model was followed by post hoc pairwise comparisons when *P*-values were significant to examine the direction and magnitude of these associations (Table 2). This model included D-12 and 6MWT, which were conducted only in hospitalised participants (*N* = 38).

**Table 2:** The main ANCOVA model with covariate age, adjusted for fixed factors, evaluated differences in the EQ-5D-5L utility score expressed by *P*-values.

EQ-5D-L utility score (*n* = 38)	Covariate (age) and fixed factors	*P*	Mean difference (95% CI) **
	**Age**	0.280	
	**Sex** Female vs. male	0.999	
	**Education** University vs. no university	**0.029**	0.115 (0.013, 0.217)
	**Smoking** Current/ex vs. never	**0.002**	0.131 (0.053, 0.209)
	**Number of persistent symptoms**	**0.004**	
	0 vs. 10		−0.003 (−0.158, 0.152)
	1–3 vs. 10		−0.153 (−0.291, −0.015)
	4–6 vs. 10		−0.141 (−0.270, −0.012)
	7–9 vs. 10		−0.189 (−0.337, −0.041)
	**Working ability based on WAI total score**	**< 0.001**	
	Poor vs. excellent		−0.283 (−0.445, −0.120)
	Good vs. excellent		−0.192 (−0.326, −0.058)
	Moderate vs. excellent		−0.097 (−0.210, 0.016)
	**Working ability based on WAI index**	**0.009**	
	Poor vs. excellent		−0.226 (−0.415, −0.037)
	Good vs. excellent		−0.011 (−0.178, 0.157)
	Moderate vs. excellent		−0.024 (−0.140, 0.092)
	**BMI in kg/m^2^**	0.531	
	25–30 vs. < 25		
	30 or more vs. < 25		
	**D_LCO_** < LLN vs. normal	0.198	
	**Comorbidity** Any vs. none	**0.017**	0.077 (0.015, 0.138)
	**D-12 total** MCID < 2.7 vs. MCID ≥ 2.7	0.468	
	**D-12 affective** MCID < 1.2 vs. MCID ≥ 1.3	**0.003**	0.257 (0.096, 0.417)
	**D-12 physical** MCID < 1.4 vs. MCID ≥ 1.3	0.815	
	**6MWT** < 80% predicted vs. ≥80% predicted	0.329	

CI: confidence interval; LLN: lower limit of normal; MCID: minimal clinically significant difference.

Bold values indicate statistically significant results (*p* < 0.05).

HRQoL was significantly affected by lower education level, poorer work ability, a higher number of persistent symptoms, more comorbidities, and experiencing more distress in the D-12 affective domain. Being a current/ex-smoker (there were only 2 of 26 current smokers in the group) was associated with a higher HRQoL than never smoking.

In the secondary ANCOVA model, we included both hospitalised and non-hospitalised participants (*N* = 59), and the model did not include D-12 or 6MWT (which were not conducted in non-hospitalised participants). HRQoL was significantly affected by poorer work ability. The number of persistent symptoms was borderline significant in this model.

The model included age, sex, smoking status, number of persistent symptoms, work ability index (WAI) total score, WAS index, BMI (body mass index), D_LCO_ (diffusing capacity of the lung for carbon monoxide), any comorbidity, as well as dyspnoea-12 (D-12) and the 6MWT. All these measures were only conducted in hospitalised participants (*n* = 38) as D-12 and 6MWT are only present for hospitalised participants and are, therefore, missing in non-hospitalised participants. Significant *P*-values were complemented with **post hoc pairwise comparisons to determine the direction and magnitude of the differences, and the results were presented as mean differences with 95% CIs.

## Discussion

Our main finding was that in the main ANCOVA model, poorer HRQoL was associated with more persistent symptoms, reduced work ability, more comorbidities, and greater affective distress 1 year after an acute episode of COVID-19. Additionally, descriptive analysis indicated that HRQoL was significantly lower in participants aged under 55 years, those on sick leave, individuals with comorbidities, including chronic pain, and those reporting persistent symptoms, especially memory/concentration problems, depression/anxiety, and fatigue. Sex and level of care at the acute phase of COVID-19 did not significantly influence HRQoL.

We showed that participants with poor or good work ability had lower EQ-5D-5L utility scores, corresponding to poorer HRQoL, than those with excellent work ability. These findings were present whether all participants or only hospitalised participants were included in the ANCOVA model, making the association between HRQoL and work ability very strong, regardless of the level of care. This finding was in line with a previous cross-sectional study on the German population, conducted on average 3–6 months after COVID-19 of varying severity, which also reported that individuals with lower HRQoL had poorer work ability (20). Also, the prospective study in 672 Swiss patients 12 months after COVID-19 revealed that lower work ability corresponded to lower HRQoL (21). A decline in work ability can bring about personal and financial difficulties and broader societal and public health implications over time. This explanation also aligns with the descriptive analysis, where participants on sick leave had significantly poorer HRQoL than those currently working in the current study.

We found that more persistent symptoms were associated with poorer HRQoL a year after COVID-19, which aligns with previous studies (22). Regarding the type of persistent symptoms, the most frequently reported by our respondents were fatigue, dyspnoea, and memory/concentration problems, which also aligns with other studies (23). Furthermore, in line with a meta-analysis on HRQoL in post-COVID-19 populations, we showed that the most commonly reported problems were in the pain and discomfort domain of the EQ-5D-5L. We also showed no difference in HRQoL between sexes and the level of care at the acute phase of COVID-19 at 1-year follow-up, similar to the prior research (16). Our findings indicate that persistent symptoms can affect individuals’ daily functioning and well-being, suggesting that persistent symptoms, rather than the acute phase severity or sex, dominate in shaping health outcomes over time.

Our study presented a significant association between lower HRQoL and affective distress. While the D-12 has not been previously validated for post-COVID-19 patients, no established MCID values exist for this population. We therefore used the MCID values from COPD studies (16). In a previous study, the D-12 affective domain was strongly correlated to anxiety and depression, as assessed by the hospital anxiety and depression scale (HADS) (24). The correlation between affective distress and HRQoL in our study confirms findings from previous studies that anxiety and emotional distress are critical issues in post-COVID-19 conditions (25).

In our study, comorbidity was associated with lower HRQoL in the descriptive analysis and ANCOVA model. This result aligns with those found in previous research (26). Our descriptive analysis showed that most pre-existing comorbid conditions, particularly chronic pain, reduced HRQoL scores compared with those without comorbidity. This suggests that underlying health status is independent in shaping HRQoL outcomes after COVID-19, regardless of current symptom burden.

We found that poorer HRQoL was significantly associated with a lack of university education. Our findings on COVID-19 infection support previous research showing lower HRQoL among individuals with lower levels of education (27, 28). In addition, previous studies report that smoking is related to poor HRQoL (29–31). In the present study, it was found that being a smoker or ex-smoker (this group consisted mainly of ex-smokers) was associated with better HRQoL as compared with never smokers. The explanation could be improving HRQoL after smoking cessation, which was shown in the previous study, where among 31 subjects who quit, the HRQoL improved within 3 months of abstinence (32). Another study used the St. George’s respiratory questionnaire (SGRQ) to assess HRQoL and reported that, at 12 weeks, the SGRQ improved significantly among the 277 participants. However, no significant differences in SGRQ scores were observed between ex-smokers and those who continued to smoke (30).

### Clinical relevance

Our study highlights the need for targeted interventions to reduce symptom burden, manage and prevent comorbidities, and support mental health to improve the HRQoL in post-COVID-19 care plans. These efforts may also positively impact work ability in patients with post-COVID-19 symptoms. This study demonstrated the association between HRQoL and work ability, confirming that both are essential predictors of general health and well-being. Future research should explore longitudinal interventions to mitigate the impact of persistent symptoms and promote recovery in these populations. Several studies have shown that persistent symptoms may change over time (13). However, there are few studies on how HRQoL progresses over time in post-COVID-19 conditions. From a public health perspective, understanding the long-term impact of COVID-19 on quality of life is crucial for planning healthcare resources, rehabilitation strategies, and social support systems.

### Strengths and limitations

The strength of our cross-sectional study was that it was one of the few that evaluated HRQoL in individuals with a laboratory-confirmed COVID-19 infection after an extended follow-up period of 1 year. Data were reported using PROMs and objective measures, such as lung function and the 6MWT. PROMs EQ-5D-5L, WAI, and D-12 have been validated in several studies in Swedish settings (9, 16). Furthermore, the survey collected a variety of COVID-19-related variables, allowing us to distinguish between different aspects of how COVID-19 has affected people. Additionally, ANCOVA was applied to adjust for potential confounders, enhancing the robustness of the results.

This study had several limitations. This single-centre study involved a relatively small number of participants. Recruitment was limited to all eligible patients in one department during the study period, and participation was voluntary rather than randomised. As a result, we were unable to compare respondents to non-respondents, which may introduce selection bias and should be considered when interpreting the results. The results of the ANCOVA analyses should be interpreted carefully, as we cannot completely rule out potential instability in the model due to variable selection. Multicollinearity among included factors of interest may have influenced some of the observed associations. Additionally, we did not control for residual confounding factors, such as wave/variant, treatment, and vaccination, which are likely to remain and should be considered when interpreting our findings.

Furthermore, some of the non-hospitalised participants were affected by COVID-19 in the pandemic’s first wave (Alpha variant), and the hospitalised participants in the second wave (Delta variant). This could have affected the HRQoL due to differences in healthcare availability, testing, vaccination strategies, and general knowledge about COVID-19 between the two waves. In this study, we present a primary analysis restricted to hospitalised patients and a secondary analysis including all patients, allowing for a comparison of the level of care.

Data collection occurred 1 year after the first infection, but we lack information on subsequent infections or vaccination status. The inclusion criteria could have biased the study results, for instance, by focusing on dyspnoea in non-hospitalised participants. Some data were collected retrospectively, such as work ability before the infection. Participants were not asked if they had experienced further COVID-19 infections within that year or whether their symptoms fluctuated over time. Additionally, the MCID used for D-12 has not been validated for post-COVID-19 conditions.

Further significant limitations include the absence of a control group to compare the HRQoL scores with individuals who did not suffer from COVID-19 during the study period. Unfortunately, we could only compare HRQoL scores before and during COVID-19, as no follow-up measures were available.

## Conclusions

Our main findings were that, a year after COVID-19, lower HRQoL was associated with more persistent symptoms, poorer work ability, comorbidities, and elevated affective distress (D-12). Our study highlights the need to address HRQoL in the rehabilitation of patients with post-COVID-19 symptoms. The study underscores the importance of work ability for HRQoL, suggesting a central role of occupational health physicians, alongside other disciplines such as primary care physicians and psychologists, in the recovery process for the growing group of patients affected by post-COVID-19 symptoms. Recognising and addressing persistent quality-of-life impairments following COVID-19 is essential not only for individual well-being but also for broader public health planning.

## Data Availability

Data can be made available on demand.
